# Blocking estrogen-induced AMH expression is crucial for normal follicle formation

**DOI:** 10.1242/dev.197459

**Published:** 2021-03-19

**Authors:** Ren Tanimoto, Kiyono Sekii, Kanako Morohaku, Jianzhen Li, David Pépin, Yayoi Obata

**Affiliations:** 1 Department of Bioscience, Tokyo University of Agriculture, 1-1-1 Sakuragaoka, Setagaya-ku, Tokyo 156-8502, Japan; 2 Department of Agriculture and Life Sciences, Shinshu University 8304 Minami-Minowa-mura Kamiina-gun, Nagano 399-4598, Japan.; 3 Department of Surgery, Harvard Medical School, Boston, MA 02115, USA; 4 Pediatric Surgical Research Laboratories, Department of Surgery, Massachusetts General Hospital, Boston, MA 02114, USA

**Keywords:** Alpha-fetoprotein, AMH, Estrogen, Estrogen receptor, Follicle formation, Mouse

## Abstract

In mammals, primordial follicles assembled in fetuses or during infancy constitute the oocyte resources for life. Exposure to 17beta-estradiol and phytogenic or endocrine-disrupting chemicals during pregnancy and/or the perinatal period leads to the failure of normal follicle formation. However, the mechanisms underlying estrogen-mediated abnormal follicle formation and physiological follicle formation in the presence of endogenous natural estrogen are not well understood. Here, we reveal that estrogen receptor 1, activated by estrogen, binds to the 5′ region of the anti-Mullerian hormone (*Amh*) gene and upregulates its transcription before follicle formation in cultured mouse fetal ovaries. Ectopic expression of AMH protein was observed in pregranulosa cells of these explants. Furthermore, the addition of AMH to the culture medium inhibited normal follicle formation. Conversely, alpha-fetoprotein (AFP) produced in the fetal liver reportedly blocks estrogen action, although its role in follicle formation is unclear. We further demonstrated that the addition of AFP to the medium inhibited ectopic AMH expression via estrogen, leading to successful follicle formation *in vitro*. Collectively, our *in vitro* experiments suggest that upon estrogen exposure, the integrity of follicle assembly *in vivo* is ensured by AFP.

## Abstract (precis)

**Highlighted Article:** An *in vitro* study inferring that successful follicle assembly under the circulating physiological level of estrogen in the mouse fetus is achieved by liver-produced alpha-fetoprotein.

## INTRODUCTION

Primordial germ cells (PGCs) generated during embryonic development subsequently form mature gametes. In mammals, whether PGCs initiate oogenesis or spermatogenesis is determined by gonadal somatic cells (McLaren, 1988). Once oogenesis is activated, all PGCs/oogonia in the cysts enter meiosis (Borum, 1961). Thereafter, oocyte cyst breakdown occurs, in which a large number of oocytes are lost and surviving oocytes are enclosed with granulosa cells, leading to serial primordial follicle assembly in the mouse ovaries in the perinatal stage (Pepling, 2012). The primordial follicles are preserved as the sole oocyte resource and their numbers continue to decrease throughout life (Pepling and Spradling, 2001). *In vitro* systems for reconstituting oogenesis are expected to help expand gamete resources, and contribute to research aimed at elucidating the unknown mechanisms of oogenesis and folliculogenesis.

Various types of abnormal follicle structures have been reported to date (Pepling, 2012). One of these abnormalities is the formation of a multiple-oocyte follicle (MOF). Deletion of *Notch2* in granulosa cells or its ligand-encoding gene *Jag1* in oocytes causes MOF, resulting in poor fertility (Trombly et al., 2009; Vanorny et al., 2014; Xu and Gridley, 2013). Activin, a member of the transforming growth factor (TGF) beta superfamily, promotes primordial follicle formation, and its natural inhibitor follistatin inhibits oocyte cyst breakdown and primordial follicle formation (Bristol-Gould et al., 2006; Kimura et al., 2011). Furthermore, pharmacological analyses showed that exposure of female fetuses or pups to estrogens, such as 17beta-estradiol (E2), genistein, the synthetic estrogen diethylstilbestrol, or endocrine-disrupting factors, causes MOF formation, accompanied by inhibition of oocyte cyst breakdown in the mouse and rhesus monkey (Chen et al., 2007; Hunt et al., 2012; Iguchi et al., 1986; Jefferson et al., 2002). Another type of anomaly is that clearly visible compartment structures similar to a normal follicle are not formed. *Foxl2*-deleted female mice fail in the differentiation of functional granulosa cells. Furthermore, granulosa cells, a continuous laminin layer, and theca cells do not regularly surround each oocyte, ultimately ceasing oocyte growth (Uda et al., 2004). Thus, follicle assembly is a fundamental event for preserving female fertility, yet the mechanisms ensuring this process remain largely unclear.

We previously demonstrated that mouse fetal gonads cultured in medium containing fetal bovine serum (FBS) exhibited MOF and/or an absence of follicular compartmentalization despite the development of a large number of growing oocytes. As a result, secondary follicles could not be isolated from these ovaries (Morohaku, 2019; Morohaku et al., 2016, 2017a). RNA-seq analysis showed that the estrogen-signaling pathway is excessively activated in *in vitro*-differentiated ovaries compared with *in vivo*-derived ovaries before follicle formation. Furthermore, we found that during follicle formation, FBS replacement with serum protein substitute (SPS), or addition of the estrogen receptor (ESR) antagonist ICI 182,780 (ICI) to the medium, dramatically increased the number of isolated secondary follicles (Morohaku et al., 2016). These results suggest that FBS contains ligands for ESRs and induces MOF and/or the absence of follicular compartmentalization *in vitro*. However, the concentration of E2 in FBS (Table S1) is much lower than in the mouse fetal and maternal serum in the late gestational stage (Dutta et al., 2014). This raises the issue of why endogenous estrogen does not inhibit normal follicle formation *in vivo*. We hypothesized that natural anti-estrogenic factors, such as ICI, *in vitro* play a role in successful follicle formation *in vivo*. The most likely candidate factor is alpha-fetoprotein (AFP) produced in the fetal liver. AFP captures estrogen and interferes with estrogen action in the fetal brain (Bakker et al., 2006; Gabant et al., 2002; Uriel et al., 1976). However, the function of AFP during follicle assembly has not yet been elucidated.

This study was conducted to identify the estrogen-signaling pathway that inhibits normal follicle formation and to determine how estrogen signaling is repressed during follicle formation *in vivo*. We used an *in vitro* model to examine whether an endogenous anti-estrogenic factor is involved in normal follicle formation *in vivo*.

## RESULTS

### Blocking estrogen signaling during follicle formation recovers the delay in oocyte cyst breakdown *in vitro*

To understand whether malformation of secondary follicles, such as MOF in ovaries cultured with alpha-minimum essential medium (α-MEM) containing FBS (basal medium), was rooted in abnormal oocyte cyst breakdown, the number of single oocytes and oocytes in the cyst was assessed in explants from days 7 to 13. Fetal ovaries at embryonic day (E)12.5 were cultured in basal medium with or without ICI from days 5 to 11 (Fig. S1).

**Fig. 1.**
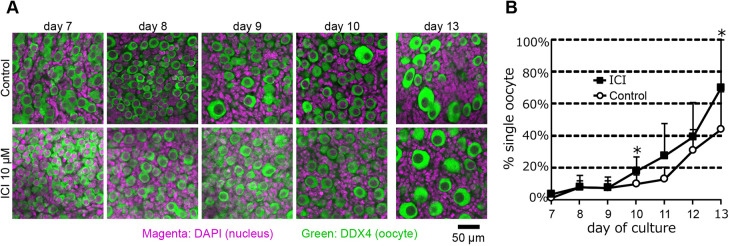
**Sequential analysis of oocyte cyst breakdown in the cultured ovaries.** (A) Immunostaining images representative of oocyte cyst breakdown in ovaries cultured with basal (upper panels, control) or ICI-containing media (lower panels). DDX4 is an oocyte-specific marker. DDX4^+^ cells are shown in green, and DAPI was used for counterstaining (magenta). (B) Sequential changes in the percentages of single oocytes in the cultured ovaries from days 7 to 13. Oocytes were counted using 1-µm *z*-stack fluorescent images of the cultured ovaries. Circles and squares indicate the mean percentage of single oocytes in ovaries cultured with basal (control, *n*=6) and ICI-containing media (*n*=5 or 6), respectively. Data are mean±s.d. Statistical significance was analyzed using an unpaired two-tailed Student's *t*-test. *P<0.05.

There was no significant difference in the percentage of single oocytes per total oocytes between ovaries cultured in the ICI-containing medium and those cultured in basal medium (control) by day 9 (Fig. 1). However, the percentage of single oocytes differed on days 10 and 13 (*P*<0.05). More than 70% of the oocytes were divided into single oocytes among the ovaries cultured in an ICI-containing medium, whereas half of the oocytes remained to connect neighboring oocytes among the ovaries cultured in the basal medium (control) on day 13. Because of the lack of estrogen receptors in non-growing oocytes (Veselovska et al., 2015; Pan et al., 2005), both estrogen and ICI should act on pregranulosa cells. This indicates that oocyte cyst breakdown was delayed in ovaries cultured in basal medium and that blocking of estrogen signaling from pregranulosa cells by ICI improved delayed oocyte cyst breakdown (Fig. 1; Tables S2, S3).

### ESR1 mediates abnormal follicle formation *in vitro*

**Fig. 2.**
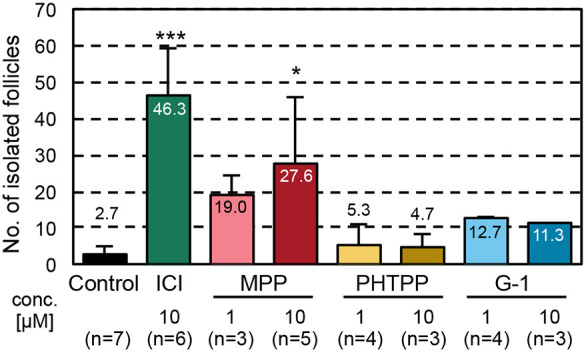
**Effects of ESR antagonists on secondary follicle formation in the cultured ovaries.** Secondary follicle formation was determined by evaluating the numbers of isolated secondary follicles from the cultured ovaries on day 17. The bars indicate the mean number of isolated secondary follicles from single ovaries cultured with basal (control, black), ICI (antagonist of ESR1 and ESR2, an agonist of GPER1, green), MPP (antagonist of ESR1, red), PHTPP (antagonist of ESR2, yellow) or G-1 (an agonist of GPER1 blue)-containing media. Data are mean±s.d. Multiple comparisons with the control were performed using Dunnett's test. *P<0.05, ***P<0.001.

ICI is an antagonist of ESR1 and ESR2, and is an agonist of G protein-coupled estrogen receptor 1 (GPER1). Ovaries cultured in ICI-containing medium recover from MOF and/or an absence of follicular compartmentalization (Morohaku et al., 2016). To assess which receptor mediates abnormal follicle formation, we added the ESR1 selective antagonist MPP, the ESR2 selective antagonist PHTPP, or the GPER1 selective agonist G-1, to the basal medium between days 5 and 11, and the number of isolated secondary follicles from the ovaries was examined on day 17 (Fig. S1). Among them, the effect of MPP was comparable with that of ICI, and it significantly increased the number of yielded secondary follicles compared with the control culture (*P*<0.05, Fig. 2). Incorporation of PHTPP or G-1 resulted in the isolation of only a small number of secondary follicles (Fig. 2). These results indicate that estrogen, or estrogen-like substances, in FBS mainly bound to ESR1, which induced abnormal follicle formation accompanied by a delay in oocyte cyst breakdown.

### Expression of anti-Mullerian hormone negatively correlates with secondary follicle isolation efficiency

**Fig. 3.**
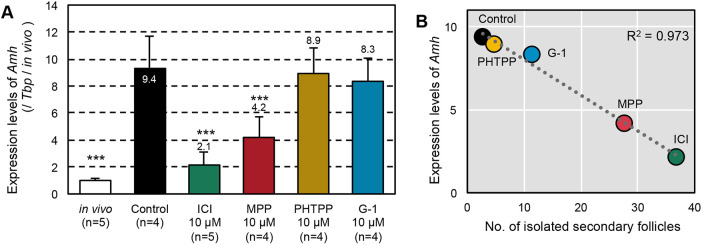
**Effects of ESR antagonists on *Amh* expression levels in the cultured ovaries.** (A) *Amh* expression levels in the cultured ovaries on day 7. The bars indicate relative expression levels of *Amh* in the ovaries of P0 mice (white) and those cultured in basal (control, black), ICI (antagonist of ESR1 and ESR2, an agonist of GPER1, green), MPP (antagonist of ESR1, red), PHTPP (antagonist of ESR2, yellow) or G-1 (an agonist of GPER1, blue)-containing media. *Tbp* was used as an internal control. Data are mean±s.d. Multiple comparisons with the control were performed by Dunnett's test. ***P<0.001. (B) Plot showing the ratio of the mean number of isolated secondary follicles on day 17 of culture to the mean *Amh* expression level in the cultured ovaries on day 7. The correlation coefficient was R^2^=0.973. *P*<0.01.

To explore the candidate genes responsible for abnormal follicle formation, we performed gene ontology (GO) analysis using differentially expressed genes in fetal ovaries cultured in basal medium on day 7 and ovaries from mice at postnatal day (P)0 (Morohaku et al., 2016). GO analysis revealed significant enrichment of the GO terms gonad development, reproductive system, and reproductive structure development for 19 genes (Fig. S2, Table S4). In particular, we focused on anti-Mullerian hormone (*Amh*), which is expressed in Sertoli cells of the testes and granulosa cells of the growing follicles (Kano et al., 2017; Kobayashi and Behringer, 2003). *Amh* showed more than tenfold higher expression in ovaries cultured in basal medium on day 7 than in ovaries from P0 mice (Table S4). Quantitative reverse transcription-polymerase chain reaction (qRT-PCR) analysis on day 7 of culture revealed that the expression level of *Amh* was significantly decreased in ovaries cultured in ICI- or MPP-containing medium and ovaries from P0 mice, compared with ovaries cultured in basal medium (control) (*P*<0.001, Fig. 3A). There was a negative correlation between *Amh* expression levels on day 7 and the number of isolated secondary follicles on day 17 (Fig. 3B, R^2^=0.973, *P*=0.00182). These results indicate that ESR1 mediated ectopic *Amh* expression and abnormal follicle formation *in vitro*.

### ESR1 binds upstream of the *Amh* transcription start site in cultured ovaries

**Fig. 4.**
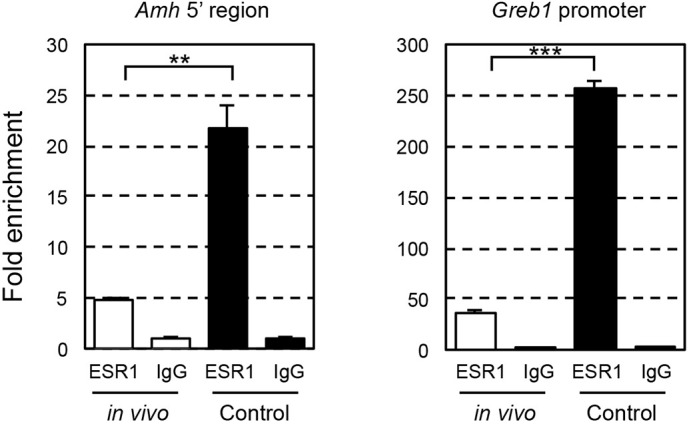
**Binding of ESR1 to the *Amh* 5′ region in the ovaries.** White bars and black bars indicate relative *Amh* amount of ESR1 ChIP to IgG ChIP in ovaries from P2 mice and ovaries cultured in basal medium for 9 days, respectively (left). *Greb1*, known as estrogen-responsive gene, is used as positive control for ESR1 ChIP (right). Data are mean±s.d. Significant differences were analyzed using an unpaired two-tailed Student's *t*-test. **P<0.01, ***P<0.001.

Ligand-activated ESR1 binds to DNA sequences known as estrogen response elements (EREs; typical sequence: 5′-AGGTCANNNTGACCT-3′), which induces changes in gene expression (Klinge, 2001). We explored EREs from −10 to +12 kb of the *Amh* transcription start site. EMBOSS analysis identified 17 sites as ERE candidates. Additionally, 19 candidate sites containing shorter (half) ERE sites (5′-AGGTCA-3′ or 5′-GACCT-3′) were examined. Preliminary chromatin immunoprecipitation (ChIP)-PCR experiments using juvenile mouse ovaries showed that ESR1 bound to the ERE half site at −92 bp (site 2). We then prepared the chromatin from ovaries cultured in basal medium for 9 days and from ovaries of P2 mice. ChIP-qPCR analyses showed that ESR1 was enriched by more than fourfold at site 2 in ovaries cultured in the basal medium compared with that of *in vivo*-derived ovaries (Fig. 4, *P*=0.00568), suggesting that ligand-activated ESR1 directly induced ectopic *Amh* expression in the cultured ovaries.

### Ectopic expression of AMH in pregranulosa cells is involved in abnormal follicle formation

**Fig. 5.**
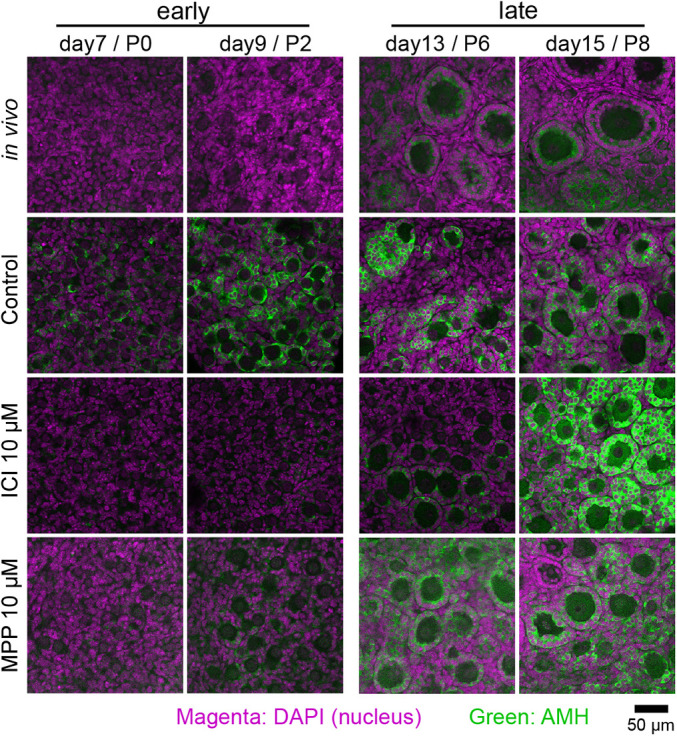
**Immunostaining analysis of AMH in cultured ovaries.** AMH (green) was detected in ovaries cultured in basal medium from day 7 (control) but not in ovaries from P0 and P2 mice. ICI or MPP incorporation largely reduced premature expression of AMH. Nuclei (magenta) were counterstained with DAPI.

We analyzed AMH protein expression in ovaries cultured in basal (control), ICI- and MPP-containing media between days 7 and 15, and in ovaries from mice at the corresponding age (P0–P8) (Fig. 5). AMH was highly expressed in the granulosa cells of the growing follicles, which appeared in the ovaries of P6 and older mice, but not in those before the emergence of primary follicles. In contrast, in ovaries cultured in basal medium (control), AMH was prematurely expressed in gonadal somatic cells and presumptive pregranulosa cells on days 7 and 9, albeit no signals from the ovaries of P0 and P2 mice were detected (Fig. 5). This ectopic expression of AMH declined in ovaries cultured in ICI- and MPP-containing media (Fig. 5). Based on these results, AMH was prematurely expressed in presumptive pregranulosa cells by ligand-activated ESR1 before oocyte cyst breakdown. This would cause abnormal follicle formation *in vitro*.

**Fig. 6.**
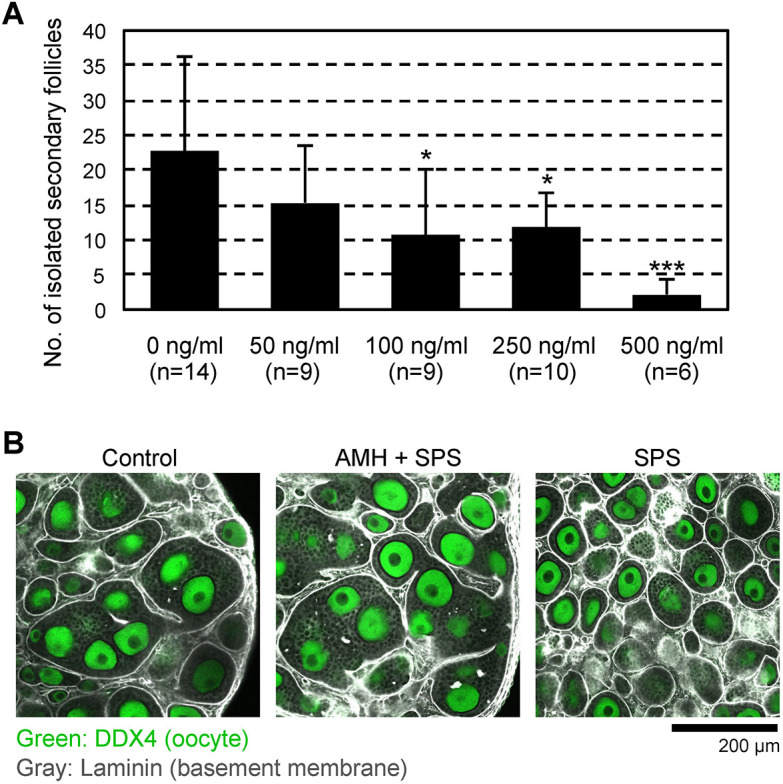
**Effects of AMH on secondary follicle formation in cultured ovaries.** (A) Efficiency of secondary follicle isolation from cultured ovaries on day 17. The bars indicate the mean number of isolated secondary follicles from single ovaries cultured with SPS-containing medium with or without AMH. Data are mean±s.d. Multiple comparisons with control ovaries cultured with SPS-containing medium without AMH (control) were performed by Dunnett's test. *P<0.05, ***P<0.001. (B) Immunostaining of follicle basement membrane and oocytes using laminin (gray) and DDX4 (green) antibodies, respectively. The ovary cultured with SPS-containing medium without AMH forms individual follicle basement membrane (right panel). Ovaries cultured with SPS and 500 ng/ml AMH-containing medium (middle panel) showed a similar phenotype to ovaries cultured with basal medium (left panel, control).

To obtain direct evidence that AMH inhibits follicle formation, we added 0, 50, 100, 250 and 500 ng/ml of AMH to α-MEM containing 10% SPS rather than FBS only from days 5 to 11 (Fig. S1), and then assessed the number of isolated secondary follicles from the ovaries on day 17. The number of isolated secondary follicles was decreased in a dose-dependent manner. When 100 ng/ml or more AMH was added to the medium, the efficiency of follicle isolation was significantly worse (0 versus 100 or 200 ng/ml, *P*<0.05; 0 versus 500 ng/ml, *P*<0.001; Fig. 6A). The laminin-immunostaining analysis showed an abnormal follicle basement membrane, which did not entirely include single follicles (Fig. 6B). Thus, ectopic expression of AMH was not only a marker of abnormal follicle formation but also a cause of this abnormality characterized by the absence of follicular compartmentalization *in vitro*.

### AFP contributes to the mechanism of blocking endogenous estrogen action during follicle formation

**Fig. 7.**
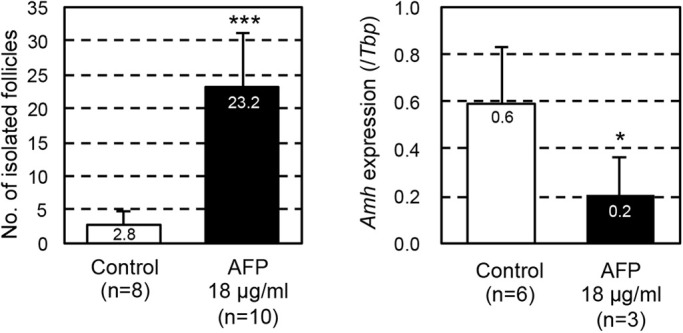
**Effects of AFP on secondary follicle formation and *Amh* expression in cultured ovaries.** The left graph indicates the efficiency of secondary follicle isolation from the ovaries cultured in basal medium (control, white bar) and AFP-containing medium (black bar) on day 17. The right graph indicates relative expression levels of *Amh* in ovaries cultured in basal medium (control, white bar) and AFP-containing medium (black bar) on day 7. Data are mean±s.d. Significant differences were analyzed using an unpaired two-tailed Student's *t*-test. *P<0.05, ***P<0.001.

Finally, we analyzed the effect of AFP on follicle formation *in vitro*. Fetal ovaries were cultured in a basal medium to which 18 µg/ml AFP was added only from days 5 to 11 (Fig. S1). The number of isolated secondary follicles on day 17 was significantly increased upon AFP addition (*P*<0.001, Fig. 7). *Amh* expression was repressed in the ovaries upon AFP addition on day 7, as expected (*P*<0.05, Fig. 7). The AFP concentration in the mouse serum was 2.7 mg/ml at P0 (Fig. S3). This suggests that AFP is an integral component of normal follicle formation *in vivo*, and endocrine factors from organs other than the ovaries are required for successful follicle assembly.

## DISCUSSION

We used an *in vitro* model to identify the estrogen-signaling pathways that inhibit normal follicle formation and to determine how the estrogen signal is repressed during follicle formation *in vivo*.

Normal follicle assembly is a prerequisite for establishing the oocyte pool in the ovaries and preserving female fertility. Just before follicle assembly, pregranulosa cells migrate into adjacent oocytes in the cyst, and oocyte cyst breakdown occurs (Pepling and Spradling, 2001; Wang et al., 2017). Defects in oocyte cyst breakdown induce abnormal follicle formation, such as MOF, and reduce fertility (Vanorny et al., 2014). Therefore, oocyte cyst breakdown is the first visible step toward normal follicle formation. Exposure to an excess of estrogen or estrogen-like substances has been shown to inhibit oocyte cyst breakdown and induce MOF in the mouse and rhesus monkey (Chen et al., 2007; Hunt et al., 2012; Iguchi et al., 1986; Jefferson et al., 2002). In contrast, estrogen was also shown to promote follicle assembly in hamsters and baboons (Wang and Roy, 2007; Zachos et al., 2002). Although the process of follicle formation is complex and heterogenous among animals, we focused on the mechanisms of estrogen action in follicle formation in the mouse.

We initially investigated whether a failure in secondary follicle formation in ovaries cultured in the FBS-containing basal medium was preceded by a delay in oocyte cyst breakdown. As expected, oocyte cyst breakdown was delayed in ovaries cultured in basal medium (control) compared with those cultured in the ICI-containing medium. The abnormal phenotype was evident on day 10 (Fig. 1). Thus, ESR ligands in FBS, namely estrogen, activated ESR, causing a delay in oocyte cyst breakdown. Although E2 could not be detected at high levels in the FBS used in this study (Table S1), unknown ligands for ESRs may exist in FBS and mouse fetal serum. In contrast, cDNA chip analysis showed lack of *Esr1* and *Esr2* expression in non-growing oocytes (Pan et al., 2005). RNA-seq data in non-growing oocytes also showed that contigs encoding *Esr1* and *Esr2* were present but in truncated forms lacking the DNA-binding domain (Veselovska et al., 2015). Therefore, estrogen does not appear to directly act on oocytes in the cyst to inhibit oocyte cyst breakdown.

Phytoestrogens, such as genistein, induce a delay in oocyte cyst breakdown and MOF formation via ESR2 (Jefferson et al., 2002). MOF induced by genistein administration was observed in wild-type and *Esr1*-deleted mice but not in *Esr2*-deleted mice. In contrast, Chen et al. (2009) reported that natural estrogen, E2, inhibits oocyte cyst breakdown and follicle assembly via ESR1. In this study, we did not obtain clear evidence that ESR2 and/or GPER1 inhibited normal follicle formation in cultured ovaries, which may be due to differences in the affinity of ligands and ESRs.

In contrast, we revealed that ESR1 mainly mediates abnormal follicle formation in ovaries cultured in basal medium (Fig. 2). Interestingly, we found that ligand-activated ESR1 bound to *Amh* and upregulated *Amh* transcription in ovaries cultured in FBS-containing basal medium. To date, there has been a clear evidence gap regarding the relationships of estrogen, AMH and follicle formation. A previous study showed that an ERE is located in human *AMH* and that the exogenous human *AMH* promoter is activated by extra estrogen (Guerrier et al., 1990); however, the effects of estrogen-induced AMH production on follicle formation are unknown. Here, we found a clear negative correlation between the *Amh* expression level before follicle assembly and the number of secondary follicles (Figs 3, 5, 6, 7). AMH inhibited normal follicle formation in the cultured ovaries (Fig. 6). This inhibitory effect is consistent with previous reports (Nilsson et al., 2011). Thus, one of the cascades of estrogen signaling underlying abnormal follicle formation was elucidated.

AMH is a member of the TGF-β superfamily. During embryogenesis, AMH is essential for masculinization and plays roles in Mullerian duct regression (Kano et al., 2017; Kobayashi and Behringer, 2003). AMH is also expressed in the granulosa cells of growing follicles and is indispensable for the appropriate maintenance of the primordial follicle pool after puberty (Durlinger, 1999; Kano et al., 2017). Although targets of the AMH signaling pathway are largely unknown in the ovaries, ectopic expression of AMH before follicle assembly can alter the developmental program in pregranulosa cells and result in abnormal follicle formation.

Exposure to extra estrogen during the perinatal stage has detrimental effects on female fertility. However, endogenous estrogen circulates in the fetal serum. A previous report showed that E2 levels in the fetal mouse serum are tenfold higher than in maternal serum after 17 days of gestation (Dutta et al., 2014). However, this estrogen never induces premature AMH expression or failure of follicle formation *in vivo*. Endogenous estrogen in the fetus is produced by the placenta, brain and ovary (Blomquist et al., 1993; McCarthy, 2008; Raunig et al., 2011; Sato et al., 2015; Tata et al., 2018; Terada et al., 1984; Weniger et al., 1993), and these organs express 3-beta hydroxysteroid dehydrogenase and aromatase (Abbaszade et al., 1997; Tata et al., 2018). Therefore, we hypothesized that natural anti-estrogen factors exist during follicle assembly *in vivo*. AFP, a major serum protein produced by the fetal liver and visceral endoderm of the yolk sac (Nayak and Mital, 1977), binds to estrogen to interfere with its activity (Jacobson et al., 1990). Previous studies demonstrated that homozygous deletion of *Afp* does not cause embryonic lethality but induces anovulation after puberty with gonadotropin-releasing hormone neuron dysfunction in female mice (Gabant et al., 2002). AFP captures locally produced E2 converted from testosterone in the female fetal brain, preventing masculinization (Bakker et al., 2006). However, the role of AFP during follicle assembly is unclear.

**Fig. 8.**
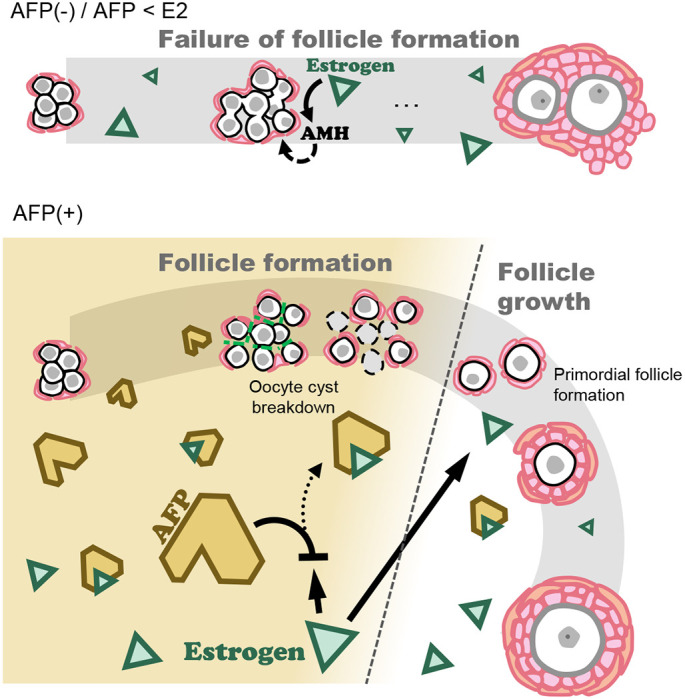
**Schematic diagram illustrating normal follicle assembly *in vivo*.** Estrogen circulates in the fetal serum during oocyte cyst breakdown and follicle assembly (Dutta et al., 2014). If estrogen binds to ESRs, AMH is prematurely expressed in pregranulosa cells in the ovaries, which in turn delays oocyte cyst breakdown and causes failure of normal follicle assembly. However, AFP also circulates in the fetal serum at a much higher level than E2. AFP captures E2, thereby inhibiting the ESR signaling pathway and/or modulating estrogen action. When follicle assembly is nearly accomplished, AFP disappears. Once primordial follicles enter the growth phase, E2 produced by growing follicles contributes to various biological processes.

In this study, we showed that adding AFP to basal medium led to successful secondary follicle formation in cultured ovaries by circumventing premature *Amh* expression (Fig. 7). This indicates that estrogen, which is present in FBS, can be captured by recombinant AFP, thereby inhibiting estrogen action. Previously, we demonstrated that the incorporation of an aromatase inhibitor, anastrozole, does not recover abnormal follicle formation (Morohaku et al., 2017a). Therefore, estrogen produced by the ovaries *in vitro* is not the leading cause of abnormal follicle assembly. As the fetal and newborn serum contains AFP at a much higher concentration compared with that of E2 during follicle assembly (Fig. S3) (Olsson et al., 1977), circulating estrogen must bind to AFP, thereby protecting the fetal ovaries from estrogen *in vivo*. It was also reported that amniotic fluid estrogen binds to AFP (Uriel et al., 1976). Thus, AFP modulates the estrogen concentration at appropriate and/or subthreshold levels during embryonic development. This would be common among other mammalian species. In cows, during follicle assembly, which occurs at 3–4 months of gestation, the serum AFP concentration peaks at 5 mg/ml (Abe et al., 1976; Yang and Fortune, 2008). The FBS used in this study contained only 130.5 ng/ml AFP (Table S1), although the age of the FBS-originating fetuses was unknown. Furthermore, AFP in the FBS may not be biologically active due to the heat-inactivation step (56°C for 30 min) before use. Whether human AFP can bind to estrogen is controversial (Mizejewski, 2003; Nishi et al., 1991); however, another factor, such as sex hormone-binding globulin, may act as a natural anti-estrogen factor (Mendel, 1989). Alternatively, estetrol (E4) produced by the human fetal liver can modulate E2 action. E4 binds to ESR1, and E4 acts antagonistically toward the proliferation of human breast epithelial cells via E2 (Holinka et al., 2008). Our *in vitro* system provides insight into the contribution of AFP and/or another anti-estrogen factor to follicle assembly *in vivo* (Fig. 8).

In conclusion, estrogen inhibits physiological follicle formation via ESRs. Estrogen-activated ESR1 induces premature AMH expression. Thus, alteration of granulosa cell differentiation by estrogen delays oocyte cyst breakdown and abnormal follicle formation. Furthermore, the natural anti-estrogen factor AFP could repress ectopic AMH expression and suppress abnormal secondary follicle formation in ovarian explants. Our study suggests that upon estrogen circulation, AFP ensures the integrity of follicle assembly *in vivo* (Fig. 8).

## MATERIALS AND METHODS

### Animals

B6D2F1 (C57BL/6N×DBA/2 hybrid) mouse fetuses were collected from C57BL/6N mothers for ovarian culture at E12.5. B6D2F1 mice were also subjected to *in vivo* control experiments at P0, P2, P6 and P8. The mice used in this study were purchased from CLEA Japan. All procedures were approved by the Institutional Animal Care and Use Committee of the Tokyo University of Agriculture.

### Ovarian culture and secondary follicle isolation

The culture conditions are summarized in Fig. S1. Ovaries were isolated from female fetuses in L15 medium (Sigma-Aldrich). Next, fetal ovaries were cultured on Transwell-COL insert membranes (Corning). The ovarian culture was performed as described previously (Morohaku et al., 2016, 2017b). Briefly, α-MEM (Gibco, Thermo Fisher Scientific) supplemented with 10% FBS (v/v) (Gibco, Thermo Fisher Scientific) was used as the basal medium. FBS was incubated at 56°C for 30 min before use. In all culture conditions, fetal ovaries were cultured in the basal medium from days 0 to 5 and 11 to 17.

To test the antagonistic and agonistic effects of ESR on follicle assembly, ICI (Tocris Bioscience), MPP (Cayman Chemical), PHTPP (Santa Cruz Biotechnology), G-1 (Cayman Chemical) and E2 (FUJIFILM Wako Pure Chemical) were dissolved in dimethyl sulfoxide (DMSO; Nacalai Tesque) at 10–100 mM. Furthermore, mouse recombinant AFP (Flarebio Biotech) was dissolved in buffer [20 mM Tris-HCl, 0.5 M NaCl (pH 8.0)] at a concentration of 3 mg/ml. Each dissolved chemical was added to the basal medium to a final concentration of 1 µM, 10 µM or 18 µg/ml, and was used for ovarian culture from days 5 to 11. During this period, the vehicle was added to the basal medium at the same percentage as the chemicals as a control. To assess the effect of AMH on follicle assembly, recombinant human AMH was produced as described previously (Kano et al., 2017) and dissolved in PBS at a concentration of 1.2 µg/µl. AMH was added to α-MEM supplemented with 10% SPS (v/v) (CooperSurgical) at 0, 50, 100, 250 or 500 ng/ml, and was used for ovarian culture from days 5 to 11. To assess secondary follicle formation, secondary follicles were isolated from the cultured ovaries using a tungsten needle on day 17.

### Immunostaining analyses

To assess oocyte cyst breakdown, the ovaries were subjected to immunostaining of the oocyte-specific marker DDX4 at days 5, 7, 8, 9, 10, 11, 12 and 13 of culture. For AMH expression analysis, *in vitro*- and *in vivo*-derived ovaries were immunostained for AMH at days 7, 9, 13 and 15 of culture, and at P0, P2, P6 and P8. To assess the follicle basement membrane, the ovaries were subjected to immunostaining of laminin at day 17 of culture. The ovaries were fixed with 4% paraformaldehyde (w/v) in PBS (−) for 0.5–1.5 h and then washed with PBS (−) supplemented with 0.1% Triton X-100 (v/v) (Nacalai Tesque) for 1 h at room temperature. The ovaries were treated with PBS (−) supplemented with 0.1% Triton X-100 (v/v) and 5% bovine serum albumin (w/v) (Sigma-Aldrich) for blocking. The primary antibodies were diluted with blocking solution. Rabbit anti-DDX4 polyclonal antibody (Abcam, ab13840, 1:400) was used to assess oocyte cyst breakdown; mouse anti-DDX4 monoclonal antibody (Abcam, ab27591, 1:200) and rabbit anti-laminin polyclonal antibody (Abcam, ab11575, 1:200) were used for double staining of oocytes and the follicle basement membrane, respectively; and goat anti-AMH polyclonal antibody (Santa Cruz Biotechnology, sc-6886, 1:400) was used to detect AMH in the ovaries. Ovaries were incubated with the diluted primary antibody for ∼10 days at 4°C. Secondary antibodies used in the subsequent procedures were F(ab′)2-goat anti-rabbit IgG (H+L) Alexa Fluor 488 (Molecular Probes, Thermo Fisher Scientific, A11070, 1:500), F(ab′)2-goat anti-mouse IgG (H+L) Alexa Fluor 594 (Molecular Probes, Thermo Fisher Scientific, A11020, 1:500) and donkey anti-goat IgG (H+L) Alexa Fluor 488 (Molecular Probes, Thermo Fisher Scientific, A11055, 1:500). The ovaries were incubated with the diluted secondary antibody for ∼10 days at 4°C. The stained samples were mounted between two cover glasses (Matsunami Glass) with a 180-µm-thick spacer (SunJin Lab) using Vectashield with DAPI (Vector Laboratories). Fluorescent micrographs were acquired using a confocal laser-scanning microscope (Zeiss, LSM710). Images of large ovarian samples were acquired from the top and bottom.

### Analysis of oocyte cyst breakdown and counting of oocytes

*Z*-stack images were obtained at 1-µm thickness. Three-dimensional images were reconstituted from these images, and then the number of oocytes with visible nuclei was counted on each plane with a 25%, 50% and 75% *z*-position of the full height. As shown in Fig. S4, DDX4-labeled cells were defined as oocytes. To confirm whether an oocyte was connected to neighboring oocytes in the three-dimensional images, oocyte cysts were assessed by scrolling back and forth along the *z*-axis until the end of the oocyte was observed. Images were processed using ImageJ software (ver.1.48, National Institutes of Health). The percentage of single oocytes was calculated from the total number of oocytes counted in the ovary.

### GO analysis

Transcriptome datasets were obtained from DRA010141 archived in the DNA Data Bank of Japan Sequence Read Archive and used for GO analysis. StrandNGS software ver.2.1 (StrandNGS) was used for GO analysis of 547 genes that were identified to be differentially expressed between fetal ovaries cultured in basal medium on day 7 and ovaries from P0 mice (normalized signal value >5; *P*<0.05; >threefold change; Morohaku et al., 2016).

### *Amh* mRNA expression analyses

Total RNA was isolated from a single ovary on day 7 of culture or from a single ovary of P0 mice using an RNeasy micro kit (Qiagen) according to the manufacturer's protocol with a minor modification. Briefly, rather than the DNase treatment process specified by the kit, eluted RNA solution was treated with RQ1 RNase-free DNase (Promega). First-strand cDNA was synthesized from the RNAs using SuperScript III (Invitrogen, Thermo Fisher Scientific). qRT-PCR of *Amh* and *Tbp* was performed using Power SYBR Green Master Mix (Applied Biosystems, Thermo Fisher Scientific) and a QuantStudio 3 Real-time PCR system (Applied Biosystems, Thermo Fisher Scientific). Gene expression levels were normalized to that of *Tbp* as an internal control. The primers were as follows: *Amh*_F 5′-TTGGTGCTAACCGTGGACTTC-3′; *Amh*_R 5′-GCGTGAAACAGCGGGAATC-3′; *Tbp*_F 5′-ATCCCAAGCGATTTGC-3′; and *Tbp*_R 5′-GCTCCCCACCATGTTC-3′.

### ChIP-qPCR analysis

For chromatin preparation, 86 ovaries cultured in basal medium for 9 days (6.2 mg, wet weight) and 73 ovaries from P2 mice (8.3 mg, wet weight) were used. ChIP assays were performed using a MAGnifyTM ChIP System kit (Invitrogen, Thermo Fisher Scientific) according to the manufacturer's instructions, with a few modifications, such as homogenizing the samples after crosslinking and sonication to obtain sheared chromatin DNA with a focused-ultrasonicator Covaris S220 after dilution. Sheared chromatin DNA samples were prepared in 215 µl of a diluted buffer, and then 10 µl of these samples were used as the 10% input control; each 100 µl was used for immunoprecipitation with rabbit anti-ERα polyclonal antibody (Merck, 06-935; 1:100) or rabbit IgG (MAGnifyTM ChIP System kit, Invitrogen, Thermo Fisher Scientific; 1:100).

ChIP-qPCR of *Amh* and *Greb1* (positive control) of the obtained ChIP DNA was performed using Power SYBR Green Master Mix (Applied Biosystems, Thermo Fisher Scientific) and a QuantStudio 3 Real-time PCR system (Applied Biosystems, Thermo Fisher Scientific). The primers *Amh*_ERE_F (5′-CTCAGGCCTCTGCAGTTATGG-3′) and *Amh*_ERE_R (5′-AGGGACGCCCCTATCAACAC-3′) were used to amplify the genomic region containing an ERE half site present in *Amh* at 92–87 bps upstream of the transcription start site (ENSMUST00000036016.5). The positive control primer pair was as follows: *Greb1*_ERE2_F 5′-TCACCCACAGTGCTGCGAGA-3′ and *Greb1*_ERE2_R 5′-GCCCTTGACCGAGGAGATGA-3′ (Lin and Lei, 2016).

### AFP and E2 measurements

Mouse AFP in the P0 mouse serum and bovine AFP levels in FBS used in this study were measured in duplicate using enzyme-linked immunosorbent assay kits (R&D Systems, for mouse; Cloud-Clone, for cow) according to the manufacturer's instructions. For E2 measurement in FBS, ethyl acetate was added to FBS or standard E2 (Kanto Chemical) solution dissolved in methanol (Kanto Chemical), and then mixed and centrifuged at 16,000 ***g*** for 3 min at 4°C. This step was repeated three times. These supernatants containing E2 were combined and dried under a nitrogen stream. The solid was reconstituted in ethanol (Kanto Chemical) and subjected to liquid chromatography-tandem mass spectrometry (Nexera UHPLC; API 4000, AB SCIEX).

### Statistical analysis

Significant differences in the percentage of single oocytes were examined using an unpaired two-tailed Student's *t*-test. For analysis of the ChIP-qPCR results and effects of AFP on follicle formation, significant differences were examined by two-tailed Student's *t*-test. Dunnett's test was performed for multiple comparisons in follicle isolation, and gene expression analyses were performed using the multicomp package in R software (ver.4.0.0). All data are mean±s.d.

## Supplementary Material

Supplementary information
