# Supplementary material for: Blocking estrogen-induced AMH expression is crucial for normal follicle formation
Source: Development. 2021 Mar 19;148(6):dev197459. doi: 10.1242/dev.197459 (PMC7990856; doi:10.1242/dev.197459)
Supplement: Supplementary information [file develop-148-197459-s1.pdf]

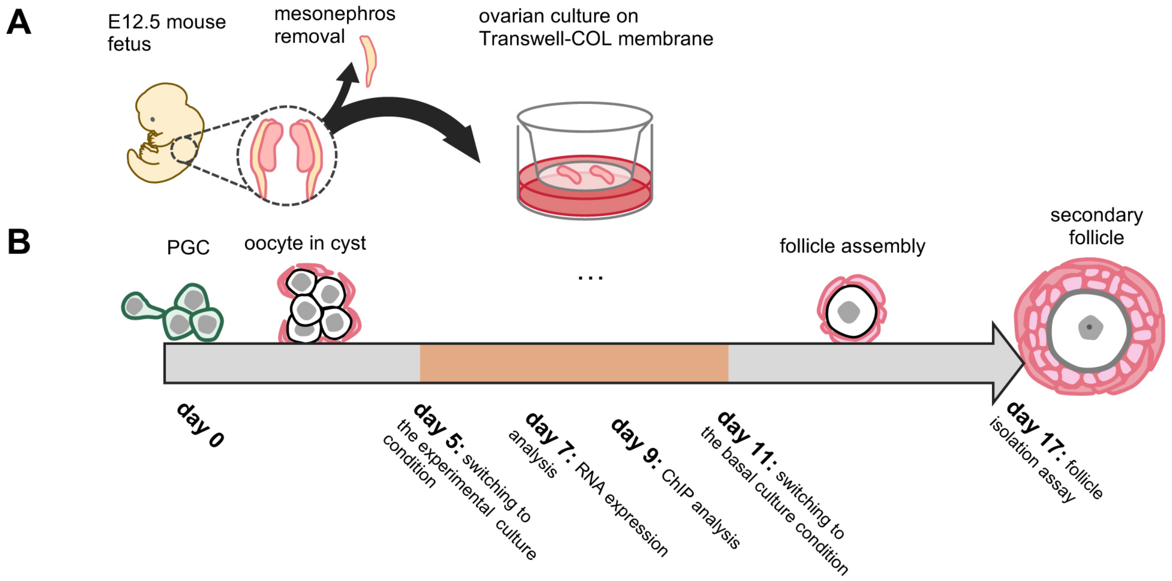


<table>
<tr><th>Treatment</th><th>days 0–5</th><th>days 5–11</th><th>days 11–17</th></tr>
<tr><td>Control</td><td rowspan="6">basal medium<br>(10% FBS + alpha-MEM)</td><td>basal medium + vehicle</td><td rowspan="6">basal medium</td></tr>
<tr><td>ICI</td><td>basal medium + 10 μM ICI</td></tr>
<tr><td>MPP</td><td>basal medium + MPP</td></tr>
<tr><td>SPS</td><td>10% SPS + alpha-MEM</td></tr>
<tr><td>SPS + AMH</td><td>10% SPS + alpha-MEM + AMH</td></tr>
<tr><td>AFP</td><td>basal medium + AFP</td></tr>
</table>

**Fig. S1. Schematic illustration of the experiments.**
(A) Procedure for ovarian culture. Embryonic day (E) 12.5 female mouse ovaries were collected and cultured on Transwell-COL membrane under various culture conditions. (B) Time course of experiments. The whole culture period was 17 days. To identify factors affecting follicle assembly, basal medium (α-MEM supplemented with 10% FBS) was switched to each experimental medium on day 5. On day 11 of culture, the experimental medium was switched to basal medium. mRNA expression analysis and ChIP-qPCR analysis were conducted on days 7 and 9, respectively. Secondary follicles from the cultured ovaries were isolated on day 17, and secondary follicle formation was assessed.

Development • Supplementary information

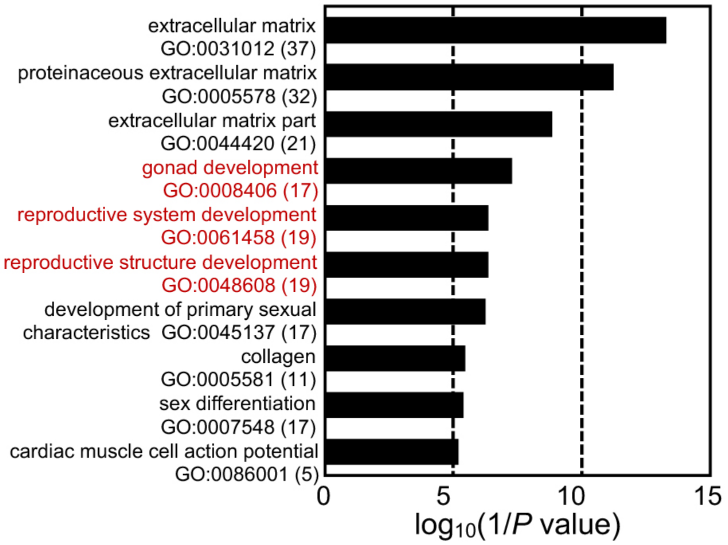


**Fig. S2. Enriched GO terms (top 10) in differentially expressed genes in ovaries cultured with basal medium compared with those from P0 mice.**

RNA-seq data were obtained from DRA010141. Significantly enriched GO terms consisted of gonad development, reproductive system, reproductive structure development, and others. The number in parentheses represents the number of genes annotated to each GO term. GO, gene ontology.

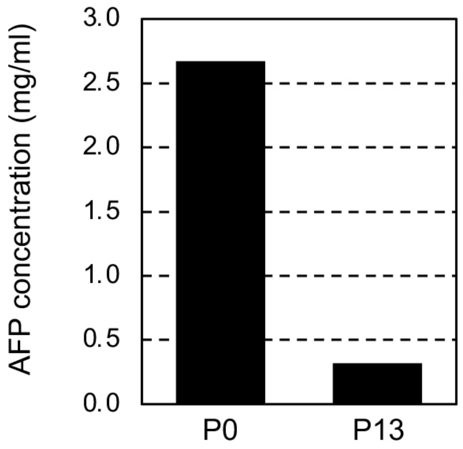


**Fig. S3. AFP levels in the serum from P0 and P13 mice.**

Development • Supplementary information

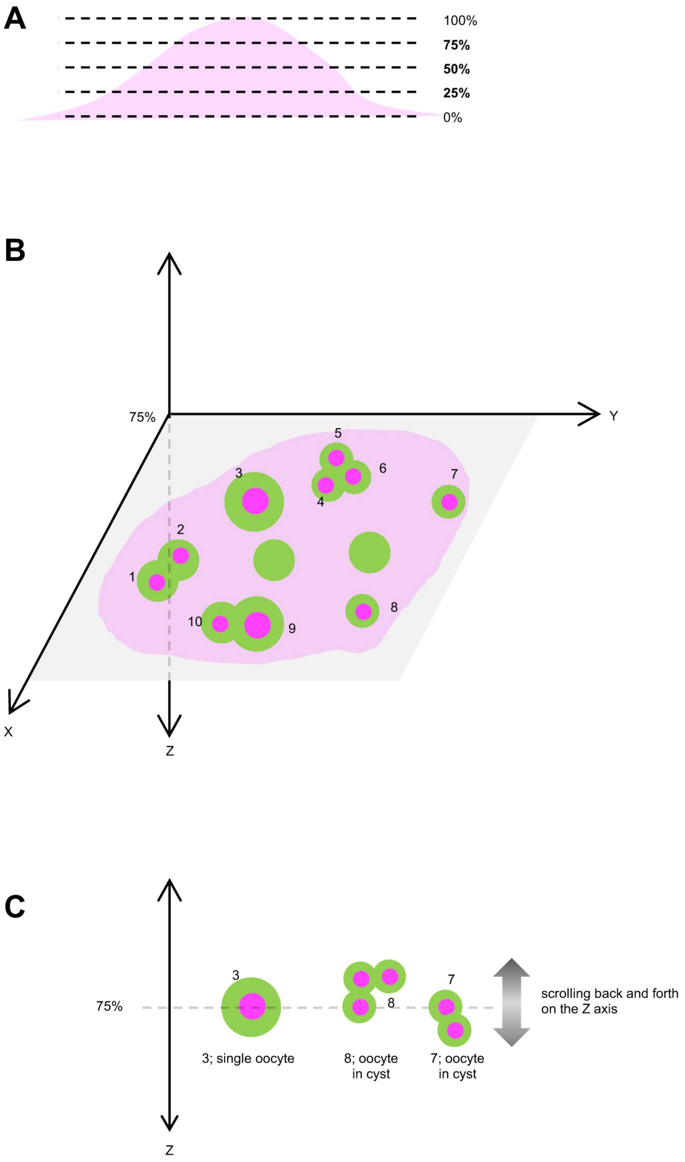


**Fig. S4. Method for counting the number of single oocytes or oocytes in cysts in the ovaries.**
Z-stack images were obtained at 1-μm thickness. (A) The number of oocytes was counted on a plane with 25%, 50%, and 75% Z-positions of the full height, respectively. (B) DDX4-labeled cells (green) with visible nuclei (magenta) were defined as oocytes. An oocyte connected with any neighboring oocyte(s) was defined as an oocyte in a cyst, whereas an oocyte that was not connected with any neighboring oocyte(s) was defined as a single oocyte in the three-dimensional images. (C) Oocyte cysts were assessed by scrolling back and forth along the Z-axis until the end of the oocyte was observed. In the example shown in this figure, a total of 10 oocytes were counted on a plane with 75% of the height. Among them, there was single oocyte identified. The percentage of single oocytes in an ovary was calculated as (total number of single oocytes on planes with 25%, 50%, and 75%)/(total number of oocytes on planes with 25%, 50%, and 75%).

Development • Supplementary information

**Table S1. Biological components in FBS used in this study.**

| Compound | Concentration |
|---|---|
| Estradiol | 8.5 pg/ml |
| alpha-fetoprotein | 130.5 ng/ml |

Development • Supplementary information

**Table S2. The percentage of single oocytes per total oocytes in ovaries cultured in basal medium (control).**

| Sample No. | day of culture | No. of oocytes | | | |
|---|---|---|---|---|---|
| | | single | in cyst | total | % of single oocytes |
| 1 | 7 | 25 | 2553 | 2578 | 1.0% |
| 2 | | 17 | 1617 | 1634 | 1.0% |
| 3 | | 16 | 1858 | 1874 | 0.9% |
| 4 | | 42 | 1272 | 1314 | 3.2% |
| 5 | | 68 | 792 | 860 | 7.9% |
| 6 | | 40 | 1503 | 1543 | 2.6% |
| | **mean** | **34.7** | **1599.2** | **1633.8** | **2.8%** |
| 7 | 8 | 34 | 1660 | 1694 | 2.0% |
| 8 | | 62 | 1310 | 1372 | 4.5% |
| 9 | | 29 | 1152 | 1181 | 2.5% |
| 10 | | 104 | 1001 | 1105 | 9.4% |
| 11 | | 78 | 1166 | 1244 | 6.3% |
| 12 | | 74 | 266 | 340 | 21.8% |
| | **mean** | **63.5** | **1092.5** | **1156.0** | **7.7%** |
| 13 | 9 | 172 | 985 | 1157 | 14.9% |
| 14 | | 17 | 1785 | 1802 | 0.9% |
| 15 | | 60 | 1508 | 1568 | 3.8% |
| 16 | | 166 | 830 | 996 | 16.7% |
| 17 | | 41 | 811 | 852 | 4.8% |
| 18 | | 42 | 1176 | 1218 | 3.4% |
| | **mean** | **83.0** | **1182.5** | **1265.5** | **7.4%** |
| 19 | 10 | 143 | 866 | 1009 | 14.2% |
| 20 | | 90 | 1329 | 1419 | 6.3% |
| 21 | | 147 | 980 | 1127 | 13.0% |
| 22 | | 43 | 464 | 507 | 8.5% |
| 23 | | 80 | 485 | 565 | 14.2% |
| 24 | | 14 | 490 | 504 | 2.8% |
| | **mean** | **86.2** | **769.0** | **855.2** | **9.8%** |
| 25 | 11 | 58 | 840 | 898 | 6.5% |
| 26 | | 66 | 418 | 484 | 13.6% |
| 27 | | 81 | 318 | 399 | 20.3% |
| 28 | | 87 | 322 | 409 | 21.3% |
| 29 | | 89 | 411 | 500 | 17.8% |
| 30 | | 69 | 719 | 788 | 8.8% |
| | **mean** | **75.0** | **504.7** | **579.7** | **14.7%** |
| 31 | 12 | 134 | 449 | 583 | 23.0% |
| 32 | | 165 | 409 | 574 | 28.7% |
| 33 | | 164 | 303 | 467 | 35.1% |
| 34 | | 184 | 318 | 502 | 36.7% |
| 35 | | 130 | 249 | 379 | 34.3% |
| 36 | | 148 | 240 | 388 | 38.1% |
| | **mean** | **154.2** | **328.0** | **482.2** | **32.7%** |
| 37 | 13 | 256 | 230 | 486 | 52.7% |
| 38 | | 223 | 329 | 552 | 40.4% |
| 39 | | 214 | 213 | 427 | 50.1% |
| 40 | | 169 | 328 | 497 | 34.0% |
| 41 | | 113 | 261 | 374 | 30.2% |
| 42 | | 127 | 33 | 160 | 79.4% |
| | **mean** | **183.7** | **232.3** | **416.0** | **47.8%** |

Development • Supplementary information

**Table S3. The percentage of single oocytes per total oocytes in ovaries cultured in the ICI-containing medium.**

| sample No. | day of culture | No. of oocytes | | | | *P*-value (v.s. Control) |
|---|---|---|---|---|---|---|
| | | single | incyst | total | % of single oocytes | |
| 43 | 7 | 20 | 1792 | 1812 | 1.1% | |
| 44 | | 39 | 1765 | 1804 | 2.2% | |
| 45 | | 36 | 1921 | 1957 | 1.8% | |
| 46 | | 115 | 1508 | 1623 | 7.1% | |
| 47 | | 71 | 2092 | 2163 | 3.3% | |
| 48 | | 68 | 1887 | 1955 | 3.5% | |
| | **mean** | **58.2** | **1827.5** | **1885.7** | **3.2%** | 0.782 |
| 49 | 8 | 80 | 809 | 889 | 9.0% | |
| 50 | | 27 | 1050 | 1077 | 2.5% | |
| 51 | | 11 | 1100 | 1111 | 1.0% | |
| 52 | | 72 | 900 | 972 | 7.4% | |
| 53 | | 131 | 812 | 943 | 13.9% | |
| 54 | | 135 | 1020 | 1155 | 11.7% | |
| | **mean** | **76.0** | **948.5** | **1024.5** | **7.6%** | 0.967 |
| 55 | 9 | 65 | 985 | 1050 | 6.2% | |
| 56 | | 27 | 925 | 952 | 2.8% | |
| 57 | | 60 | 945 | 1005 | 6.0% | |
| 58 | | 93 | 608 | 701 | 13.3% | |
| 59 | | 73 | 1091 | 1164 | 6.3% | |
| | **mean** | **65.7** | **917.1** | **982.8** | **7.0%** | 0.875 |
| 60 | 10 | 281 | 947 | 1228 | 22.9% | |
| 61 | | 238 | 850 | 1088 | 21.9% | |
| 62 | | 147 | 1051 | 1198 | 12.3% | |
| 63 | | 148 | 483 | 631 | 23.5% | |
| 64 | | 149 | 712 | 861 | 17.3% | |
| 65 | | 102 | 1150 | 1252 | 8.1% | |
| | **mean** | **177.5** | **865.5** | **1043.0** | **17.7%** | 0.037 |
| 66 | 11 | 111 | 611 | 722 | 15.4% | |
| 67 | | 121 | 662 | 783 | 15.5% | |
| 68 | | 114 | 697 | 811 | 14.1% | |
| 69 | | 240 | 188 | 428 | 56.1% | |
| 70 | | 259 | 595 | 854 | 30.3% | |
| | **mean** | **170.4** | **603.1** | **773.5** | **24.8%** | 0.273 |
| 71 | 12 | 165 | 361 | 526 | 31.4% | |
| 72 | | 192 | 609 | 801 | 24.0% | |
| 73 | | 191 | 494 | 685 | 27.9% | |
| 74 | | 283 | 266 | 549 | 51.5% | |
| 75 | | 350 | 204 | 554 | 63.2% | |
| 76 | | 180 | 197 | 377 | 47.7% | |
| | **mean** | **226.8** | **355.2** | **582.0** | **40.9%** | 0.322 |
| 77 | 13 | 200 | 80 | 280 | 71.4% | |
| 78 | | 153 | 108 | 261 | 58.6% | |
| 79 | | 314 | 90 | 404 | 77.7% | |
| 80 | | 346 | 80 | 426 | 81.2% | |
| 81 | | 273 | 38 | 311 | 87.8% | |
| 82 | | 288 | 71 | 359 | 80.2% | |
| | **mean** | **262.3** | **77.8** | **340.2** | **76.2%** | 0.025 |

**Table. S4.** Genes included in three GO terms “gonad development”, “reproductive system development”, and “reproductive structure development” using annotations for the differentially expressed gene set in *in vitro*-derived ovaries.

| Symbol | Fold Change | $log_2$ RPKM | |
|---|---|---|---|
| | | *in vitro* (Control) | *in vivo* |
| *Amh* | 11.57 | 11.4 | 7.9 |
| *Foxa1* | 6.19 | 5.3 | 2.7 |
| *Nos2* | 5.21 | 9.9 | 7.5 |
| *Frzb* | 4.23 | 6.3 | 4.2 |
| *Inha* | 3.17 | 14.4 | 12.7 |
| *Sfrp1* | -3.04 | 12.2 | 13.8 |
| *Kdr* | -3.14 | 10.7 | 12.3 |
| *Gata3* | -3.34 | 6.8 | 8.5 |
| *Lfng* | -3.64 | 6.9 | 8.7 |
| *Ccnd1* | -4.28 | 9.3 | 11.4 |
| *Srd5a2* | -4.91 | 4.5 | 6.8 |
| *Rbp4* | -4.54 | 5.9 | 8.1 |
| *Gata1* | -4.01 | 6.4 | 8.4 |
| *Sfrp2* | -4.21 | 7.4 | 9.5 |
| *Cga* | -4.67 | 4.0 | 6.2 |
| *Osr1* | -6.57 | 6.2 | 8.9 |
| *Myocd* | -6.95 | 4.0 | 6.8 |
| *Sfrp5* | -22.09 | 1.9 | 6.3 |
| *Wnt10b* | -25.56 | 1.7 | 6.3 |

Development • Supplementary information
